# Inferring Gene Family Histories in Yeast Identifies Lineage Specific Expansions

**DOI:** 10.1371/journal.pone.0099480

**Published:** 2014-06-12

**Authors:** Ryan M. Ames, Daniel Money, Simon C. Lovell

**Affiliations:** 1 Computational and Evolutionary Biology, Faculty of Life Sciences, The University of Manchester, Manchester, United Kingdom; 2 Department of Ecology and Evolutionary Biology, University of Kansas, Lawrence, Kansas, United States of America; Swiss Federal Institute of Technology (ETH Zurich), Switzerland

## Abstract

The complement of genes found in the genome is a balance between gene gain and gene loss. Knowledge of the specific genes that are gained and lost over evolutionary time allows an understanding of the evolution of biological functions. Here we use new evolutionary models to infer gene family histories across complete yeast genomes; these models allow us to estimate the relative genome-wide rates of gene birth, death, innovation and extinction (loss of an entire family) for the first time. We show that the rates of gene family evolution vary both between gene families and between species. We are also able to identify those families that have experienced rapid lineage specific expansion/contraction and show that these families are enriched for specific functions. Moreover, we find that families with specific functions are repeatedly expanded in multiple species, suggesting the presence of common adaptations and that these family expansions/contractions are not random. Additionally, we identify potential specialisations, unique to specific species, in the functions of lineage specific expanded families. These results suggest that an important mechanism in the evolution of genome content is the presence of lineage-specific gene family changes.

## Introduction

The creation of new genetic content in the form of new genes is a key component of genome evolution. New genes can arise through a variety of mechanisms including gene duplication, retroposition, horizontal gene transfer and *de novo* origination [1]; however, gene duplication has been recognised, since the 1970s, as the most prevalent source of new genes [2]. Indeed, in a comparison of the relative contributions of these gene creation mechanisms, gene duplication was shown to have produced 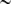
80% of new genes in several *Drosophila* species [3].

Rates of gene gain by duplication have been shown to be high in a variety of species, and it has been suggested that in eukaryotes 50% of genes are expected to duplicate at least once every 35–350 million years [4]. In *Drosophila* the rate of gene gain has been estimated to be in the range of 5 to 11 genes every million years [3] and as high as 17 genes every million years [5]. However, current genome content is a balance between the rate of gene gain and the rate at which genes are lost. Indeed, the most common fate of duplicate genes is expected to be nonfunctionalisation [4], which may be followed by removal from the genome. That the size of the genome appears to be constant over time is probably due to the high rate of gene gain coupled with a high rate of gene loss. It is therefore important to consider the rates of both gene gain and gene loss independently in order to accurately understand genome evolution.

Reconstructing histories of gene families is currently an active area of research, and there have been several methods developed, along with some genome-wide studies of the evolution of gene families [6]–[11]. Inferences of gain and loss events along the human lineage of the mammalian phylogeny suggests that, between humans and chimpanzees, the complement of genes differs more than the sequences of orthologous nucleotides [12], leading to the argument that a “revolving door” of gain and loss leads to large differences between the genomes of humans and chimpanzees. Likewise, in comparisons of gene families in several species of *Drosophila* [5] large numbers of genes are both gained and lost with over 40% of families varying in size. Genome-wide studies of gene family evolution have also been conducted in species sets containing yeast [13]–[15]. In all species analysed a large turnover of genes is seen, and specific functions are associated with these changes.

Many different methods have been developed to study gene family evolution. Novel reconstruction algorithms [16], [17] have been used to infer the histories of gene families. These algorithms a incorporate gain, loss and horizontal gene transfer and utilise phyletic profiles and a species tree. Parsimony [13] and weighted parsimony [9] have both been used to infer the evolution of gene families. Although these methods are quick and can be applied to genome-wide studies, they cannot account for multiple events (gain and subsequent loss) on a single branch.

As an alternative to parsimony methods that make use of phyletic profiles and a single species tree there are many methods that make use of gene trees produced from protein families. These methods aim to reconcile the gene trees with the single species tree and in the process of reconciliation infer the gain and loss events. Several software tools have been developed for gene/species tree reconciliation [18]–[22]. As with the parsimony methods these tools are quick, can be applied to whole genomes and have been shown to be accurate, although they may be affected by bias in some cases [23], [24]. More recently, a range of reconciliation methods [6], [8], [15], [25], [26] have used probabilistic models or Bayesian methods to infer the gene trees and for gene/species tree reconciliation. These methods have shown to be both accurate and applicable to whole genome analyses.

The problem of inferring gene family histories has also been addressed with the development of likelihood-based methods that make use of probabilistic models [7], [9], [10], [12], [14]. These methods do not rely on individual gene trees but instead use a probabilistic model to infer the evolutionary histories of gene families based on a species phylogeny and phyletic profiles for each family. These methods have been shown to be accurate, applicable to genome-wide analyses and can infer multiple events on a single branch. As these methods are dependent on an underlying model, the biological realism of these models is important for their accuracy, and so, much research is focused on producing more biologically relevant models.

We have previously produced a method with several models of gene gain and loss, allowing for the inference of gene family histories, on a whole genome level, with variable duplication branch lengths and rates of evolution among families [9]. However, the model used in our previous study did not capture all the biological complexity of gene family evolution. We have considered innovation events such as *de novo* gene gain and horizontal gene transfer that may be much more widespread than originally thought, particularly in yeast [27]. However, ourselves, and many others have not considered the complete removal of a gene family by pseudogenisation. To gain a more complete understanding of these processes more biologically accurate models are needed.

Here, we apply a new evolutionary model, implemented in DupliPHY-ML [9], to infer the evolutionary histories of gene families from 9 yeast species, using data from the Génolevures project [28]. Our model, BDIE, allows the estimation of the relative rates of gene birth (new gene gain in existing families), death (gene loss by pseudogenisation), innovation (*de novo* gene gain or horizontal gene transfer creating a new family) and extinction (loss of a complete gene family) in yeast gene families on a whole genome scale. We are able to identify families that have experienced lineage specific expansions in the yeast species and link these to specific functions. We are also able to identify functions that appear to be repeatedly associated with expanding families in multiple lineages indicating that these functions may provide common adaptations in these yeast. In addition, we can also identify functions linked with expanding families in a single lineage suggesting that lineage specific gene family evolution plays a key role in specialisation.

## Results

### Rates of gene family evolution

**Figure 1:**
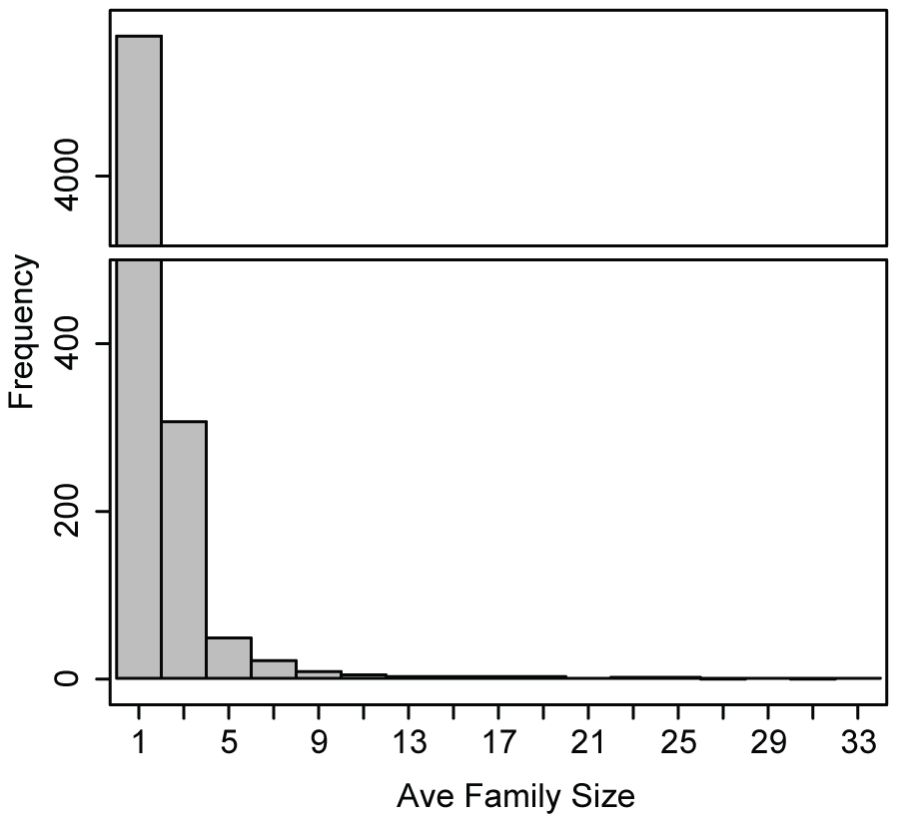
The distribution of average family sizes in the Génolevures data.

We used gene family data from the Génolevures project [28]. Here, the families represent phylogenetic groups of genes related by homology and identified by the similarity between protein-coding genes from all the species. There are a total of 4,578 families (after removal of some families - see methods) with an average size of 9.72 genes and a largest family size, in any single taxa, of 54 members. We also looked at the average number of members in each taxa per family (Figure 1), which shows that in most families the each species contains 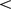
5 members. Lists of family membership can be found in File S1.

A key feature of DupliPHY-ML, is the ability to estimate the rates of gene loss (*d*), gene family extinction (*e*) and the innovation (*i*) of new gene families relative to the rate of gene gain (*b*). In this analysis the rate of gene gain (birth) refers to gene duplication in an existing gene family and gene loss (death) refers to the loss of a gene in a family with 2 or more members. Thus, extinction refers to the loss of a final member of a gene family and innovation refers to the gain of a new gene family by processes such as horizontal gene transfer or *de novo* gain. In our data set we find that *b* 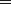
1.0, *d* 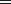
4.05, *e* 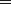
0.59 and *i* 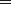
0.05. The estimates indicate that the rate of gene loss through pseudogenisation is much higher than the rate of gene duplication. This is a surprising result; although it is possible that genomes in at least some of these species are shrinking, it is perhaps more likely to indicate some artefacts from the data generation. We also find that the rate of extinction of a gene family is much lower than the rate of gene loss. The rate of new family formation is extremely low suggesting that in yeast, new gene gain by horizontal gene transfer or *de novo* gain is rare.

**Figure 2:**
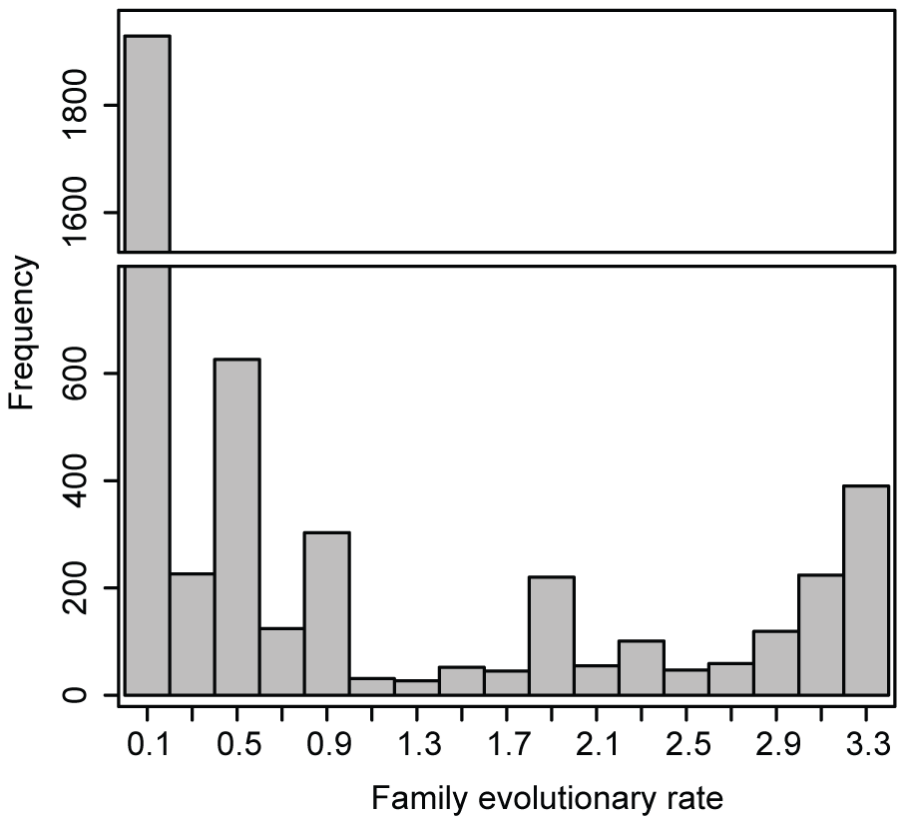
The rate of family evolution in the Génolevures data estimated by DupliPHY-ML as the mean posterior rate with gamma-distributed rates across families. Here a high estimated rate indicates a family that is evolving quickly and has undergone many duplication and loss events in its history. The average rate is 1.

Using DupliPHY-ML to infer the ancestral history of gene families, estimates of ancestral family sizes are shown in File S2. DupliPHY-ML also allows us to infer how fast or slow individual families are evolving in terms of gains and losses over the phylogeny. For example, families with a fast evolutionary rate will have had many duplication and loss events in their inferred history. We see that the majority of families are evolving very slowly with only a minority of families displaying rapid evolution (Figure 2).

Data quality may affect the estimation of these rates. It is apparent that both the rates of gene loss and extinction are relatively high compared to the rate of gene gain. If some of the genome sequences for these species are not complete then this missing data may be construed as loss by DupliPHY-ML, which would act to erroneously increase the relative rates of loss and extinction. Indeed, the majority of gene families are very small (Figure 1) and regions with zero sequence coverage in a subset of species may erroneously appear to be extinction events. The effect of such missing sequences would lead to an increase in the extinction rate. However, we note that the rate of gene loss is much higher than that of extinction suggesting that even with these potential effect of missing data, extinction still seems rare compared to gene family contraction.

### Validating the estimated rates of gene family evolution

As the estimated rates of gain and loss may be affected by both the model and the data used we attempted to validate our results. We see that on the Génolevures data our BDIE with gamma-distributed rates across families (+G) we estimate a very high rate of gene loss. In order to check that this is not a consequence of our model choice we repeated the analysis using our birth-death-innovation model [9]. The estimated rates for all the models can be found in Table S1. When we compare BDIE+G and BDI+G we see that the estimates of extinction are similar at 0.05 and 0.08 for the BDIE+G and BDI+G models respectively. The rate of death for the BDIE+G model is higher than that for the BDI+G model. This is likely due to the addition of the extinction parameter. As we think that extinction is a relatively rare event (only changes in size from 1 to 0 cause innovation and all other family size decreases are explained by death), and these rates are averaged over all families, by including extinction we remove rare events from this average, and as a consequence, the death rate increases.

We see similar patterns when we compare the the BDIE and BDI models. As with the gamma models the estimated rate of death is higher in the BDIE model for the reasons discussed above. The estimated rates of innovation are similar, 0.48 and 0.70 for BDIE and BDI respectively. Interestingly, in the non-gamma models there is a large increase in the estimates of the rate of innovation. We have seen that under the gamma model small families and families where one or more species has zero members tend to evolve faster. Our data also consists of many small families. Since the innovation parameter in the BDIE model describes going from 0 to 1 gene in a family, we expect that the innovation parameter will have the greatest effect on smaller families. This suggests that small families may need to be handled differently, which is not possible with the BDIE and BDI models. Further study of this result is warranted as it is suggestive of current models being misspecified.

In order to to test the effect of clustering parameters on the estimates of the rates of birth and death we generated 4 new gene families sets using Tribe-MCL [29] and a range of values to affect cluster granularity. The estimated rates for each clustering can be found in Table S2. As we increased the cluster granularity we identified more gene families and the average size of the gene families decreased. We find that with decreasing family size we see an increase in the estimated rate of death. We speculate that this may be due to the fact that as the death rate increases relative to the birth rate the stationary distribution of family size becomes more skewed towards smaller families and so better fits the distribution we see with larger granularity.

In order to quantify the effect of data quality on our results we generated a new set of gene families excluding *S. cerevisiae*, which has the highest quality sequence with the most annotated genes. When we repeat the DupliPHY-ML analysis we find that *b* 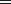
1.0, *d* 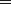
2.89, *e* 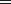
0.25 and *i* 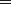
0.08. The relative rates estimated without *S. cerevisiae* change from those estimated using all the species. Most notably, the rates of gene loss and extinction are reduced indicating that there is a higher rate of gene gain inferred in this analysis compared to our original analysis. Although the values of these estimates change, the overall pattern of these estimates remains the same.

We have used several methods of validating the estimated rates of gene family evolution. In each case by altering the gene family evolution model or the input data we see variation in the estimated rates. In many cases this variation can be explained. Importantly, in all cases the trends of the results remain the same; The rate of gene loss is higher than the rate of gene birth, the rate of extinction is lower than that of birth and innovation appears to be a rare event.

### Gene family evolution over the phylogeny

**Figure 3:**
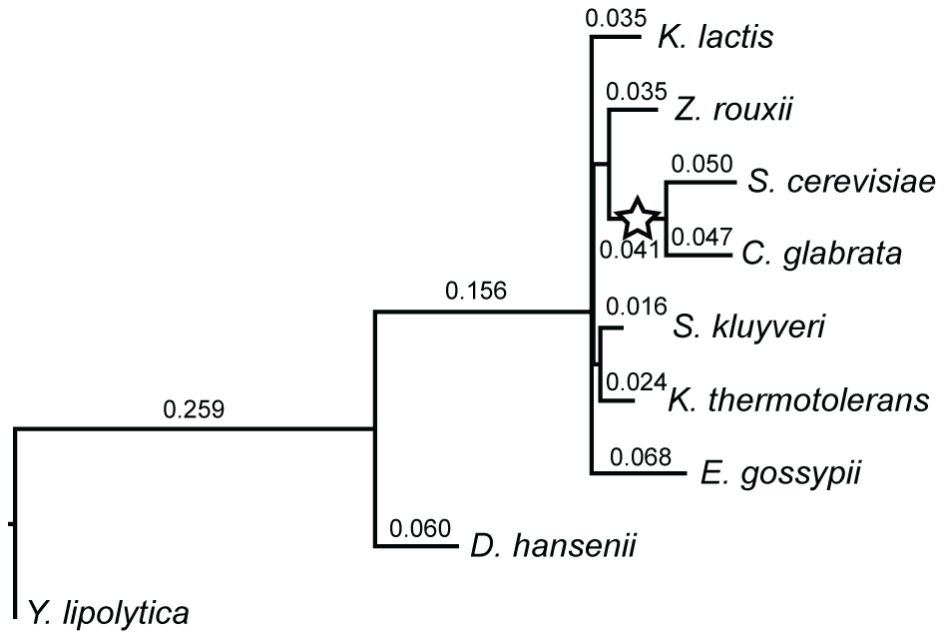
The phylogeny for the Génolevures data. Branch lengths represent the average number of gain and loss events per gene family as inferred by DupliPHY-ML. The branch on which the whole-genome duplication occurred is indicated with a star.

DupliPHY-ML estimates the average number of gain and loss events per gene family on each branch. These events, represented as branch lengths, are useful for identifying lineages, including ancestral lineages, that have a high turnover of genes in gene families. It should be noted that model misspecification may lead to very short internal branches followed by longer branches at the tip of the tree. In the species phylogeny (Figure 3) we see a long internal branch after divergence from the outgroup Y. *lipolytica*. Notably, Y. *lipolytica* has a branch length of zero suggesting there are no lineage specific events on this branch. This is unlikely to be an accurate estimate, since Y. *lipolytica* would be expect to have at least a few branch-specific events. The long internal branch length from the outgroup indicates a large turnover of genes on this branch and recapitulates the larger genetic distance between the out groups and more closely related species in the tree. Other long internal branches may represent expansions or contractions of gene families that are common to multiple species within the phylogeny. There are short branch lengths leading to *S. kluyveri* and K. *thermotolerans* (Figure 3), suggesting that these species have a very similar evolutionary history and have not had much lineage-specific evolution of gene families.

Terminal branches with long lengths represent lineage specific turnover of genes in gene families. Species with long branches may represent many gains of genes in a family and/or many losses. *D. hansenii* and *E. gossypii* both have longer terminal branches than the other species, indicating a large turnover of genes in these species. Since gains, and potentially losses, of genes in families may indicate some adaption to a new environment or evolution after speciation. In order to identify signals of environmental adaptation we first need to identify significant lineage specific expansions and contractions of gene families.

### Lineage specific expansions of gene families

We used the net change in family size on each terminal branch compared with the net change on all other branches to identify those families with significant lineage specific expansion. We specifically chose the net change in order to identify those families that have undergone expansion and those genes have been retained, rather than identifying families with just a high turnover of genes. We have identified a large number of lineage specific expanding gene families (Table 1). In *S. cerevisiae* and *D. hansenii* we can identify more than 50 such families. The ranges of expanding family sizes also varies greatly within and between species. In all species with identified expanding families we observe families with only 2 members identified as expanding. In these cases it may be that all other species lack the family or have single member families (singletons) and the expansion to 2 members is lineage specific and significant by our definition.

**Table 1:** Lineage specific gene family expansions in the Génolevures data.

Taxa	Num Genes	Num families	Ave size	Min size	Max size
*S. kluyveri*	5321	19	5.21	2	24
*K. lactis*	5076	19	4.15	2	12
*K. thermotolerans*	5092	15	5.93	2	22
*Y. lipolytica*	6448	0	0.00	0	0
*C. glabrata*	5203	13	5.46	2	13
*Z. rouxii*	4991	20	6.15	2	21
*S. cerevisiae*	6663	53	6.86	2	33
*E. gossypii*	4715	12	3.50	2	6
*D. hansenii*	6272	51	7.45	3	54

The observed patterns of evolution of gene families, including both lineage-specific expansions and slowly-evolving families, may be due to either selection or neutral evolution. If the process of expansion is neutral we expect the newly introduced genes to nonfunctionalise and later be removed. If selection is acting to increase family size in specific families we would expect that these families would be associated with specific functions that may aid adaptation, and, in particular, may be relatable to known environmental adaptation routes in yeast. Additionally, in the presence of selection we expect that some of these functions may be common to multiple species. In order to determine whether selection is operating, we determined the functional classes of genes where families are changing rapidly.

In order to identify the functions relating to the lineage specific expanding gene families we used the Gene Ontology (GO) [30]. First, we identified enriched GO slim terms for each of the expanding families (File S3). We then visualised the GO slim terms on a Voronoi treemap and highlighted the enriched terms. These visualisations allow us to detect similarities and differences in types of gene families that are expanding in these species.

**Figure 4:**
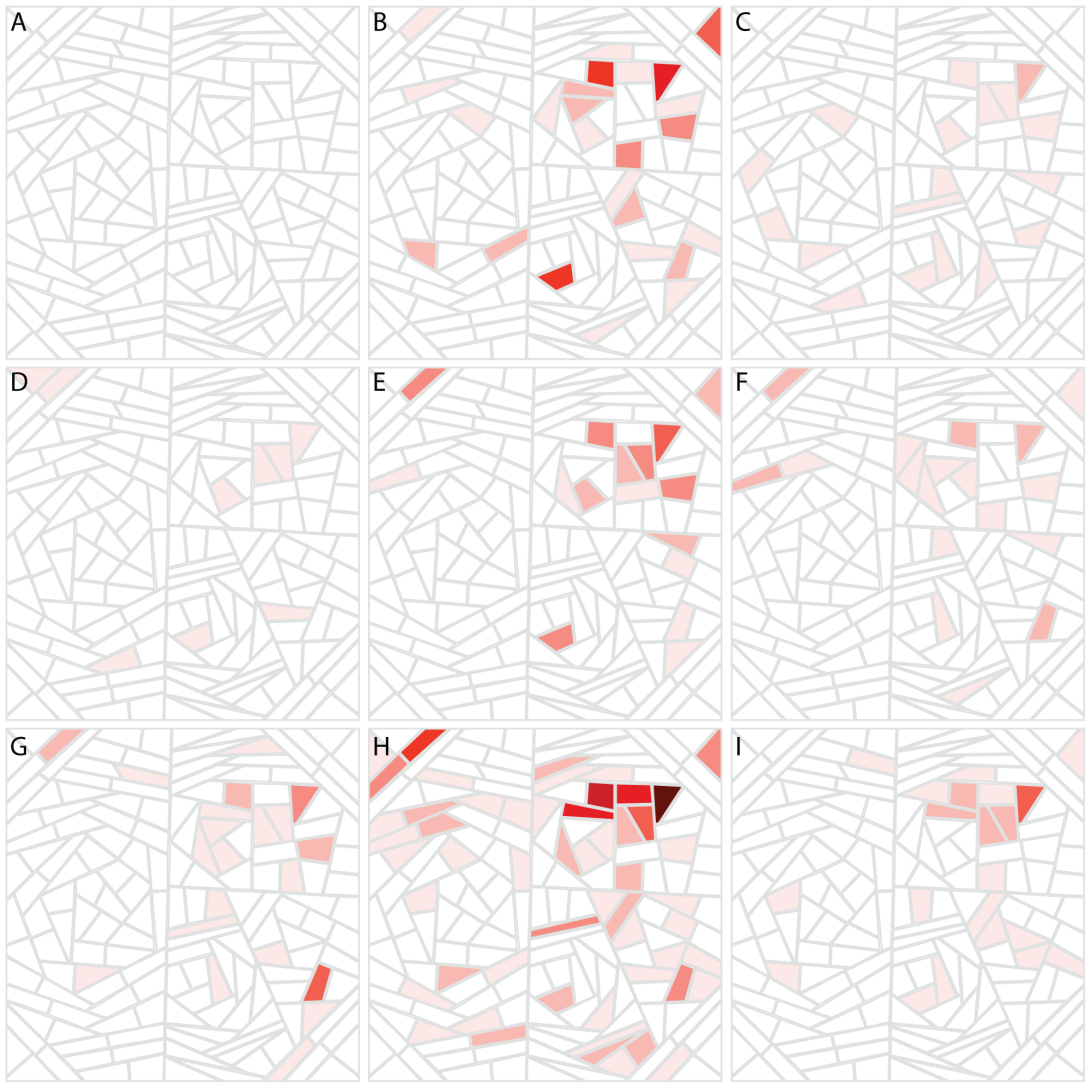
Functional enrichment of lineage specific expanding gene families for GO slim terms. Each cell represents a GO slim term from the biological process, molecular function or cellular component ontology. The positioning of the cells is determined by a term overlap metric so that more similar terms are positioned close together. Cells are shaded if one or more expanding gene family shows enrichment for that term. The intensity of the shading represents the number of gene families showing enrichment for that term. The most intense colour shows 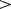 10 families are enriched. Each panel represents a species from the Génolevures data set: *Y. lipolytica* (A), *D. hansenii* (B), *E. gossypii* (C), *K. lactis* (D), *S. kluyveri* (E), *K. thermotolerans* (F), *Z. rouxii* (G), *S. cerevisiae* (H) and *C. glabrata* (I). Lists of enriched terms in expanding families are available in File S3

The species analysed show a wide variance in enrichment of GO terms (Figure 4). Most species show few enriched GO terms in expanding families. *S. cerevisiae* shows the most enrichment with some terms enriched in multiple expanding gene families. Enriched *S. cerevisiae* terms include those related to transport of amino acids and carbohydrates as well as enzyme activity such as isomerase, helicase and hydrolase activity. Transport of carbohydrates and amino acids are commonly enriched GO terms and are also enriched in the expanding families of *D. hansenii* and K. *thermotolerans*. Finally, we can identify expanding families that might be indicative of adaptation to a specific environment given the family's functional annotation; Z. *rouxii* is the only species to show enrichment for terms relating to the response to chemical stimuli and response to oxidative stress.

The observation that many of the expanding families have similar functional annotation suggests that many of the expansions we observe are driven by selection rather than random turnover of genes, or systematic gaps in sequence data. The common identification of nutrient uptake terms enriched in expanding gene families suggests that the expansion of gene in nutrient transporter families may be a common response to new environments or selection pressures. In this scenario following speciation which may have been precluded by a change in environmental conditions, selection for more efficient or varied uptake of nutrients from the environment may result in the observed expansions. In contrast, the identification of families showing unique functional annotation, such as the response to oxidative stress in Z. *rouxii*, suggests that we are also able to detect species specific adaptations.

Interestingly, we can identify rapidly expanding families of unknown function. As not all genes from these species can be annotated with *S. cerevisiae* identifiers, our data contain many genes with no known functions. By annotating these genes with an “unknown” function, we can test to see whether genes with this unknown function are enriched in any rapidly expanding families. Indeed, we find enrichment for unknown functions in all species except *S. cerevisiae* and Y. *lipolytica*, which has no rapidly expanding families. The presence of these expanding families suggests that there are species specific adaptations using novel functions. We also identify two families enriched for the unknown function that are rapidly expanding in multiple species. Family GL3C0410 is enriched for the unknown function in *C. glabrata*, *D. hansenii* and *S. kluyveri*. Family GL3R0575 is also enriched for the unknown function in *D. hansenii* and *K. lactis*. The same family expanding in multiple species may indicate that the function represented by these families is important to multiple species.

### Lineage specific contractions of gene families

In addition to looking at families showing rapid expansion we also looked at families showing significant contraction along a terminal branch. Contraction of gene families may signify the removal of non-adaptive functions or a streamlining of the genome and may result from specific adaptations. We find fewer families experiencing significant contraction than families experiencing expansion. *E. gossypii* has the largest number of contracting families with 28. Interestingly, *D. hansenii*, which has more than 50 expanding families only contains a single significantly contracting family suggesting the *D hansenii* genome has recently undergone an increase in size.

As before we used the GO to identify the functions of the contracting gene families. We find several terms common to multiple species again suggesting that species specific losses may reflect common adaptations. *S. cerevisiae*, *E. gossypii*, *S. kluyveri* and K. *thermotolerans* all show contractions of gene families related to the golgi apparatus. Furthermore, *C. glabrata*, *E. gossypii*, *S. kluyveri*, K. *lactis* and Z. *rouxii* all show enrichment for transmembrane transport terms in contracting families. Full lists of the GO terms associated with contracting families can be found in File S4.

### Whole-genome duplication

Two of the species in our data set (*S. cerevisiae* and *C. glabrata*) have a whole-genome duplication (WGD) in their evolutionary past [31], [32]; the branch on which this event occurred is indicated by a star on Figure 3. Subsequent to this duplication, a large number of duplicate genes were lost [32], [33]. In common with all previously-published methods, our BDIE model does not incorporate any parameters that represent whole genome duplication. The presence of whole-genome duplication therefore has the potential to give rise to biases in the results, especially since the subset of genes retained after the WGD is enriched for specific functions [33], [34].

There are three possible patterns of gene loss that may have occurred subsequent to WGD. First, a gene duplicated by WGD may be lost in both daughter species; in this case neither the duplication nor loss would be present in our input data. Second, a gene duplicated by WGD may be retained in both daughters. DupliPHY should assign this duplication to the WGD branch, leading to a large number of gains, and so long branch length in Figure 3. Third, a duplicated gene may be differentially lost in one daughter species and retained in the other. In this case the only signal for the duplication is the presence of a gene in one species. Here the duplication event may be assigned to the terminal branch rather than the ancestral WGD branch and then inferring a loss in the other species.

The first case is invisible to all methods of ancestral reconstruction that are based on analysis of extant genomes, including ours. However, we see that on the WGD branch of the phylogeny 4,161 gene families do not appear to change size. Since, by definition, all gene families must change size during a WGD, this figure gives an indication of how many events are invisible to our method and similar methods. We see some evidence of case two (retention in both species). There are 285 gene gains (in 263 families) on the WGD branch, and only 172 losses (in 152 families). This pattern is the reverse of that seen throughout the tree as a whole, where gene loss is four times more likely than gene gain.

In order to investigate the possible causes of bias that arise from case 3 (differential retention being miss-assigned), we determined how many of the gains on the *S. cerevisiae* and *C. glabrata* lineages have arisen via WGD. We used the assignment of duplications to either the WGD set (data from Byrne and Wolfe [35]) or small-scale duplication set (SSD, data from Hakes *et al* [36]). We find that, for *S. cerevisiae* 64 of the lineage specific gene gains arise from the WGD event, 203 from SSD events, with 97 gains unknown. For *C. glabrata* only 2 genes arise from WGD, 23 from SSD and 46 genes arise from unknown origins. The rapidly expanding families that contain WGDs for both *S. cerevisiae* and *C. glabrata* can be found in File S5. As the method of Hakes *et al* is conservative in assigning SSDs and much research has focussed on identifying WGDs, we believe that the majority of the “unknown” gains are likely to be SSDs. Although it is still possible that some of these genes may be unannotated WGDs or may have arisen from innovation events such as horizontal gene transfer between species. Since there are many more WGDs than SSDs (551 and 272 gene pairs, respectively), the observed pattern is unlikely to be an artefact arising from differing number of duplications in the input set. We conclude, therefore, that although the whole-genome duplication must have affected the pattern of observed duplications, particularly for the *S. cerevisiae* and *C. glabrata* lineage-specific duplications, the effect is unlikely to change our conclusions substantially.

In addition we also repeated the inference of duplicate gene family histories excluding any gene families in the Génolevures data that contained a known WGD from Byrne and Wolfe [35]. This data set contained 4,064 families with an average size of 8.88 genes. Here we find that *b* 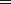
1.0, *d* 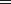
5.09, *e* 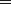
1.01 and *i* 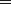
0.05. Overall the relative rates show the same pattern as the original analysis. The rate of loss is high compared to duplication and innovation is rare. Notably, the relative rates of both gene loss and gene family extinction have increased compared to the original analysis. This suggests that most genes retained from the WGD were inferred as gains in our analysis and the removal of these families has increased the estimation of the rate of loss and extinction relative to the rate of gene gain.

## Discussion

Here we have used the software DupliPHY-ML to infer the evolutionary history of gene families in a range of yeast species and present evidence that suggests lineage specific gene family expansions lead to species-specific functional adaptations. We have implemented a new model with parameters for birth, death, innovation and extinction.

We note that in our model the birth parameter represents the retention of a gene after duplication rather than simply the occurrence of a duplication event. The observed birth rate is therefore a combination of the underlying rate of duplication, and the various factors that affect fixation rate, including selection. Although we may define the biological event represented by birth differently from previous work the model is similar to that of many other birth-death models used to study the evolution of gene families [6], [8], [14]. We make this distinction because it may be expected that the underlying duplication rate would correlate with gene family size, since large families contain more genes that are candidates for duplication. However, there is clear evidence for selection to remove or silence recently duplicated genes [4]. The combination of these factors means that the size of families are not well described by a simple model where birth rate increases linearly with family size [37], [38]. There is, however, a weak correlation between the rate of change and family size; this conclusion holds for a range of species, including yeast. It is therefore likely that our model could be improved by the addition of a suitably weak correlation parameter.

DupliPHY-ML estimates the rate of gene loss, gene family extinction and gene family innovation relative to the rate of gene gain. The rates we estimate from the data show that the rate of gene loss is much higher than the rate of gene gain. Pseudogenisation followed by gene loss is thought to be the most common fate of duplicate genes [39]. The rate of innovation is much lower than that of the rate of gene gain by duplication within an existing family. Indeed, *de novo* gain of genes seems to be very rare, with only a handful of reported cases in yeast [40], [41], *Drosophila* [3] and human [42]. We identified innovation events in our data by looking for examples of gene family gain within our phylogeny *i.e.* going from 0 to 1 or more members along a branch. The gene *BSC4* has been reported as a *de novo* gene in *S. cerevisiae* and has been associated with DNA repair during the stationary phase in *S. cerevisiae* when shifted to a nutrient-poor environment [41]. Here we find it is a member of a singleton family, and therefore, as arising from an innovation event. Hall *et al.* [40] identified two genes, *BDS1* and *URA1*, in *S. cerevisiae* that originated by horizontal gene transfer. In our data we also identify *BDS1*, an aryl- and alkyl-sulfatase gene required for the use of specific sulfates as sulphur sources, as arising from an innovation event. Interestingly, this gene appears to be present in both *S. cerevisiae* and *K. thermoltolerans*, suggesting that this gene may have been introduced to these genomes by separate horizontal transfer events. *URA1*, a dihydroorotate dehydrogenase involved in the in the *de novo* biosynthesis of pyrimidines, is not identified in our data as originating from an innovation event as all species studied here contain a gene with similarity to *URA1*, meaning the gene family was present at the root of our phylogeny.

Recently, Carvunis *et al* [27] described a process of gene formation from non-genic sequences through the formation of proto-genes that later evolve into genes. The finding of a significant number of proto-genes (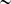 1900) in the yeast genome suggests that the rate of innovation identified in this study may be well below the true level of *de novo* gene gain. Interestingly, we find evidence of 1, 292 innovation events across all 8 species used in this study. We point out that although are estimates of the rate of innovation are much smaller than that of gene duplication we still find a large number of cases of innovation in our data. Additionally, it is possible that the exclusion of singleton families from this analysis has led to the low estimate of the rate of innovation of genes.

As with innovation, we find that gene family extinction is rare compared to the loss of a single gene in a family with more than one member. In mammals the rate of extinctions is lower than both expansions and contractions [12]. It has been suggested that gene family extinction may occur when there are shifts in nutrient availability, meaning specialised families are no longer necessary for growth [43].

We note that the estimated rates of death and extinction appear to be artificially high. Although the pairwise comparisons of the rates of birth/innovation and death/extinction make biological sense there are alternative explanations for the high estimates of death and extinction. Firstly, there are differences in the set of genes identified in these species that may have arisen because of low sequencing coverage or errors in gene identification. We have demonstrated that the estimated rates are not overly affected by the presence of one well sequenced species by removing *S. cerevisiae*. When *S. cerevisiae* is removed from the analysis the relative rates estimated by DupliPHY-ML are affected. In particular the estimated rate of loss is reduced from 4.05 to 2.89. These estimates of loss may be artificially high because repeated lineage specific losses of ancestral genes may inflate the estimated rate of loss. Alternatively, the genomes may be shrinking from some large ancestral genome at, or before, the last common ancestor of the set of species used in our analysis. Finally, large differences in the number of identified genes (Table 1) across all species may explain the high estimates of loss. CAFE 3 [11] attempts to correct for missing data but these types of corrections could not be used for this study as we have no accurate estimate of the amount of data missing from these genomes.

We have repeated much of our analysis to validate our estimates of the rates of birth, death, innovation and extinction by removing families, species, running different models and using data generated with different clustering methods. In some cases we find that the estimates of these parameters vary depending on the data or the model used. This indicates that the model used in this study still does not capture the complete biological processes of gene family evolution and suggests that further research of these models is warranted. Importantly, for all of the validation the trends in the estimates of these parameters remains the same and so, we can draw biological conclusions from our results.

Extended internal or terminal branches may represent areas of the phylogeny that have undergone accelerated gene family evolution in response to some selection pressure. In the species phylogeny *D. hansenii* and *E. gossypii* show the longest terminal branch lengths. These long branches may well be representative of adaptation to their respective environments. Indeed, these yeasts appear to occupy very specialised environments with *D. hansenii* being a cryotolerant, halotolerant marine yeast often found in cheese [44] and *E. gossypii* being a cotton pathogen [45]. Alternatively, long branch lengths may represent some stochastic turnover of genes in areas of the genome that experience high rates of gene gain or loss, such as subtelomeric regions [46], [47].

The DupliPHY-ML method allows the determination of rates of gene gain and loss in a branch-specific manner. Families with low rates of changes may be tightly constrained by selection, with changes in the numbers of members having a deleterious effect. By contrast, the fast evolving families may represent those that are expanding or contracting in response to selection from the environment. Indeed, Demuth *et al.* [12] identified a set of rapidly evolving gene families in a mammalian phylogeny and showed that these families were associated with the same biological functions as quickly evolving genes and regulatory regions. Similarly, gene families that are evolving quickly in yeast may have important biological functions if there is selection pressure from the environment to increase the membership in these families. Alternatively, these families may be experiencing stochastic turnover of genes. In order to distinguish between evolution by adaptive or neutral processes we first needed to identify gene families that show significant expansion in a lineage. Families showing significant expansion on the terminal branches of the phylogeny represent lineage specific examples and may provide insight into the genetic mechanisms that lead to species specific adaptations.

We find that the ASP family is rapidly expanding; the ASP genes are asparaginases involved in the catabolism of alternative nitrogen sources [48], [49]. This family is shown to be expanding on the lineage leading to *S. cerevisiae* which is consistent with previous analyses [50]. Other *S. cerevisiae* lineage specific expansions show an expansion of two of the major subtelomeric gene families. There are expansions in the seripauperin multigene (*PAU*) family and the *DUP240* integral membrane protein family. The *DUP240* family is a collection of nonessential genes that have been linked with membrane trafficking processes [51]. Subtelomeric regions have been shown to contain many duplicated genes that may be a consequence of higher rates of recombination in these regions [46], [47]. Interestingly, in *S. cerevisiae* some genes gained in these families have relocated to internal sites on chromosomes [50] suggesting that duplication events that increase gene family membership may be coupled with relocation events.

The use of GO [30] gives an overview of the types of genes in expanding gene families. There is a wide range of variation in functional annotation, through enrichment of GO terms, in the expanding families. Several species show very little enrichment whereas *S. cerevisiae* shows wide ranging enrichment. The species in these data are sufficiently diverged that it is possible to detect differences in the types of families that are expanding in these species (Figure 4). We can identify unique functions associated with families expanding in a single species that might indicate lineage specific adaptation. A single expanding family in the species Z shows enrichment for response to chemical stimuli and response to oxidative stress. This family is comprised of 5 genes, 3 of which have high similarity to *S. cerevisiae CTA1* and 2 with similarity to *CTT1*, suggesting lineage specific duplication of these genes. *CTA1* is a catalase A involved in hydrogen peroxide detoxification and is important during the oxidative stress response [52]. *CTT1* is a catalase T and is also involved in hydrogen peroxide detoxification and is known to be induced under oxidative stress conditions [52], [53]. In *C. albicans* it has been shown that trehalose mobilisation is important for tolerance of hydrogen peroxide [54]. Z. *rouxii* is well known to be tolerant to salt and osmotic stress [55], [56] and it has been suggested that the high expression of trehalose synthesis genes under non-stress conditions [57] may be important in a range of stress responses [58]. It may be the case that Z. *rouxii*'*s* naturally high rate of trehalose synthesis makes the species tolerant of a wide range of stresses and the expansion of a family associated with hydrogen peroxide resistance is a complementary adaptation to a specific environmental stress. Additionally, the presence of enrichment for an “unknown” GO term in expanding gene families suggests that there are also species specific adaptations using novel functions.

It is also possible to identify common functions associated with families expanding in multiple species. As these expansions are lineage specific and thus, independent, any common functional enrichment of these families may represent repeated selection for a specific adaptation. Many species contain lineage specific expanding families that are associated with carbohydrate transport and metabolism as well as amino acid transport. *S. cerevisiae* is well adapted to the uptake of sugars and has a range of transporters for different sugars [59], [60]. It seems that there has been expansion in the sugar transporter family in *S. cerevisiae* through a combination of whole genome and tandem duplication possibly to facilitate the evolution of aerobic fermentation [61], [62]. Indeed, we also see a large expansion of a sugar transporter family in *S. cerevisiae*. *D. hansenii* also shows expansion of families containing carbohydrate transporters and has been shown to be able to transport and utilise a range of sugars including hexoses and pentoses [63]. The prevalence of sugar transporter family expansion across several species of yeast may reflect a general adaptation strategy to environments containing an array of sugars with different genes amplified to fine-tune a yeast's metabolism to a specific sugar. Indeed, K. *lactis* which is found mostly in dairy products has acquired the ability to utilise lactose. We find that few *HXT* genes have been retained [62] and no expanding families show enrichment for sugar transporters.

Interestingly, we can also identify gene families that are contracting and the functions represented by these families. As with expanding families it is possible to detect different families with the same functional annotation contracting in multiple species. These functions tend to be associated with general transmembrane transporter activity and exocytosis (File S4). It has been argued that loss of genes may be adaptive [64] by the removal of non-adaptive functions and streamlining of the genome. It is tempting in this case to speculate that the losses shown here, coupled with gains of specific transporters, represent a restructuring of the uptake capabilities of the yeast in response to a new environment. Thus contractions as well as expansions may play a role in species specific adaptations.

The evolutionary histories of gene families can provide important insights into the past and present adaptations of species. We have inferred the evolutionary histories of all gene families across a variety of species, using the most sophisticated published models. We find that families evolve at a variety of rates and that these rates vary on different branches of the phylogeny. This variability suggests that rates are likely to be dictated by a range of selection pressures that act on a particular species. Additionally, we have identified families that show significant expansions on the terminal branches of the phylogeny. These families may be experiencing high rates of expansion because of neutral mechanisms or alternatively, may be under selection for increased membership. Indeed, we have identified several families showing rapid lineage specific expansion that are located in subtelomeric regions of the genome that are known to undergo regular recombination events. We also see that functional annotations associated with these expanding families suggest that there is selection for expansion of stress response and sugar transporter families. These expansions contribute to species differences and their individual functional specialisations.

## Methods and Materials

### Genomic and gene familiy data

Annotated genome sequences for 9 species of hemiascomycete yeast were downloaded from the Génolevures project [28]. These species were *Eremothecium gossypii, Candidia glabrata, Debaryomyces hansenii var. hansenii,* K*luyveromyces lactis var. lactis, kluyveromyces thermotolerans, Saccharomyces cerevisiae, Saccharomyces kluyveri,* Y*arrowia lipolytica* and Z*ygosaccharomyces rouxii*. These specific species were selected because they have previously been used to generate a set of gene families using a consistent and comprehensive method [65]. For all species we used the predicted ORFs and protein sequences identified by the sequencing project. Genes were annotated using BLAST [66] and the *S. cerevisiae* annotated genome as a reference. An E-value cutoff of 1×10^−4^ was used to call annotations in the BLAST results.

We used gene families from the Génolevures data as previously identified [65]. Briefly, the authors aligned the proteomes of the yeast species using BLAST [66] and the Smith-Waterman algorithm. Following this Tribe-MCL [29] was used to cluster the dat set using a range of inflation coefficients to produce clusters at different granularities. Finally, the authors use consensus clustering and an election algorithm to compare clusters and categorise them as robust, consensus, multiple choice or unique. In order to use the most reliable predictions of gene families we used those classified as “robust” or “consensus” families. Additionally, we removed families where any single taxa contains more than 75 members. We did this to make the analysis computationally tractable but it might also be the case that extremely large families are under specific selection pressures that may not be well defined by our current models. We note that this removes some notoriously complicated families such as polyproteins and repeat domains as well as single gene families which represent unique genes in the tree.

The specific clustering parameters used to generate the Génolevures data and our selection of “robust” and “consensus” families, may have introduced some bias into the identified families. In order to assess any bias in the data performed our own clustering on the raw Génolevures data. Here we used the protein sequences available for each species from the Génolevures website to perform an all against all BLAST search. The resulting network of BLAST hits was then clustered using Tribe-MCL [29] with a range of inflation values that control cluster granularity. Tribe-MCL was run with 4 inflation values of 1.4, 2, 4 and 6. For each of the resulting sets of gene families we ran DupliPHY-ML with the BDIE+G model (described below). We note that for computational tractability we still removed families where any single taxa contains more than 75 members and removed any singleton families. This analysis allows us to determine the effect of clustering on our results.

### Phylogenetic tree

A phyogenetic species tree is necessary to infer the evolutionary histories of gene families. DupliPHY-ML does not use gene trees to infer events but instead will infer gain and loss events on the species phylogeny. Here, we used a subset of the cladogram presented in [67] to get the phylogenetic tree structure. Branch lengths were estimated for this structure using Baseml of the PAML package [68]. We used MUSCLE [69] to align the coding sequences of 2324 common genes between all species (based on BLAST annotation described above), excluding any genes that appeared to have more than one copy in any species, and these alignments were concatenated. The list of common genes used in this step can be found in File S6. Baseml was run using the general time reversible model with no molecular clock to infer branch lengths. These branch lengths were used to test for significantly expanding or contracting gene families after the inference of gene family histories.

### Inferring the evolutionary history of gene families

Given the identified gene families and phylogenetic trees for these data sets we then used DupliPHY-ML [9] to infer the evolutionary history of each gene family. These histories were inferred under the Birth-Death, Innovation and Extinction (BDIE) model with gamma-distributed rates across families. This model is similar to the Birth-Death-Innovation (BDI) model described previously [9], with the addition of an extinction parameter. DupliPHY-ML estimates each of the parameters from the available gene family data. Here the rate of gene birth corresponds to the rate of gene gain by duplication in existing families. The rate of gene death simulates the loss of a duplicate gene in a family with more than 1 member. Innovation represents the gain of a novel gene family through *de novo* gene gain or horizontal gene transfer and extinction represents the loss of a gene family from the tree. Once a gene family has been lost, it may only arise again through an innovation event. The BDIE model has an instantaneous rate matrix, **Q**, defined by equation 1.
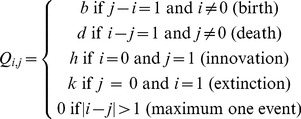
(1)


We also supplied DupliPHY-ML with a set of unobservable families. Unobservable families represent potential phyletic patterns that can occur in these species but have been purposely removed from the analysis. In this case these are families that contain only a single gene. DupliPHY-ML corrects for these cases using the method of Felsenstein [70].

In order to validate the BDIE model we repeated the analysis of the Génolevures data using the previously described BDI model [9]. We also ran these models without gamma-distributed rates across families. All models were run on the same dataset and with all other run options kept constant.

To identify lineage specific gene family expansions we compared the change in number of members of an inferred gene family on an internal node to the observed gene family size the tips of the tree. The change in the number of members was normalised by the terminal branch length to control for greater divergence time allowing for higher turnover. If this change was positive and greater than the mean expansion or contraction on all other branches in the tree we used a Wilcoxon Rank Sum test to check for a significant expansion. We controlled for false discovery rate using the method of Benjamini and Hochberg [71]. All tests with P
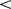
0.05 after false discovery rate correction were taken as the set of rapidly expanding families. We note that by simply calculating the change in family membership from an ancestral node to a child node is likely to underestimate the true number of changes along a branch as some genes may be gained and lost on that branch. This simplification means that the calculations of the amount of change along a branch are likely to be an underestimate and so our results may be conservative. It also means that we focus on those gene gains that have been retained in the extant species.

We also identified lineage specific gene family contractions. Much like for expanding families we looked for the change in number of family members on each branch of the tree normalised by the branch length. If the change was negative (i.e. loss) and the number of losses was greater than the mean number of events on all other branches we used a Wilcoxon Rank Sum test to check for a significant expansion. We controlled for false discovery rate using the method of Benjamini and Hochberg [71]. All tests with P
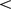
0.05 after false discovery rate correction were taken as the set of rapidly contracting families.

### Controlling for genome annotation and whole genome duplicates

The presence of genomes with better quality sequence and annotation may affect our analysis by artificially altering the estimated rates of gain and loss. In order to determine the effects of variable genome quality on our analysis we removed *S. cerevisiae* and repeated the analysis. To create a new set of gene families that exclude *S. cerevisiae* we first performed an all against all BLAST [66] search of all remaining species. We then used Tribe-MCL [29] with an inflation parameter of 1.2 to cluster the BLAST similarity network and generate a new set of gene families. Finally, we repeated the inference of gene family history using DupliPHY-ML as described above.

We also note that the new model of genome evolution (and all other existing models) doesn't have a parameter for large scale duplication events. As the phylogeny used in this study is known to contain a whole genome duplication event [32] we controlled for the effect of whole duplication on our estimates of gain and loss by removing all families that contained a known whole genome duplicate. After the removal of these families we repeated the DupliPHY-ML analysis as described above.

### Functional annotation, enrichment and visualisation of gene families

In order to assign function to the expanding gene families we used the slim version of the Gene Ontology (GO) [30]. As GO terms are associated with genes via *S. cerevisiae* identifiers we annotated all genomes with an *S. cerevisiae* identifier (see above). We note that some genes that have no GO term annotation and as a consequence some expanding gene families or some members of these families have no functional annotation. There are also cases of genes in some species having no associated gene identifier from *S. cerevisiae*, in these cases we have assigned these genes a pseudo “unknown” GO term. This allows us to identify families that are enriched for an unknown function and may perform a novel function not performed by *S. cerevisiae*. We then used the GO slim term associations available at the *Saccharomyces* Genome Database (SGD) to assign GO terms to genes.

To test for enrichment of a GO term in a gene family we used Fisher's exact test. Here, the sample was all annotated genes in a gene family and the background population was all the annotated genes in the yeast genome. The sample successes therefore were all genes in the gene family annotated with the specific GO term and the population successes were all genes in the genome annotated with the specific GO term. All P-values were false discovery rate corrected using the method described by Benjamini and Hochberg [71] with a significance cutoff of P
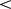
0.05. This analysis was applied to both the rapidly expanding and contracting gene families.

In order to visually compare functional enrichment, the Term Overlap (TO) metric of the GLASS software (available at http://www.bioinformatics.ic.ac.uk/glass/) was used to determine the pairwise distances between GO slim terms. A tree-structure was generated from these pairwise distances using the neighbour-joining algorithm implemented in Quicktree [72]. The tree structure was then represented in two dimensions using Voronoi Treemaps [73], [74], implemented with GLASS [75]. In this visualisation each cell represents a GO slim term, whose location within the panel is determined by the TO distance to all other terms. A cell is coloured if one of more expanding gene families are enriched for that GO term, with the intensity of the colour indicating the number of gene families enriched for that term.

## Supporting Information

File S1 This file lists all of the Génolevures families including the identified present in each family for each species.
(TXT)

File S2 The ancestral reconstructions for each family analysed with DupliPHY-ML. The first line of the file is a newick string representation of the yeast phylogeny used in the study. The rest of the file is a tab delimited list of gene family ID and the number of members present at each node of the tree.
(TXT)

File S3 The functional enrichment of GO slim terms for rapidly expanding families in each species. The file lists the enriched terms, the ontology within GO slim and the raw and corrected P-values.
(XLSX)

File S4 The functional enrichment of GO slim terms for rapidly contracting families in each species. The file lists the enriched terms, the ontology within GO slim and the raw and corrected P-values.
(XLSX)

File S5 Expanding families in *S. cerevisiae* and *C. glabrata* that contain whole genome duplicates.
(TXT)

File S6 The list of 2324 common genes used to infer the branch lengths on the yeast phylogeny.
(TXT)

Table S1 Comparison of parameter estimates of 4 DupliPHY-ML models run on the Génolevures data.
(PDF)

Table S2 The effects of gene family data on the estimation of rates under the BDIE+G model.
(PDF)
