# Supplementary material for: Inferring Gene Family Histories in Yeast Identifies Lineage Specific Expansions
Source: PLoS One. 2014 Jun 12;9(6):e99480. doi: 10.1371/journal.pone.0099480 (PMC4055711; doi:10.1371/journal.pone.0099480)
Supplement: Table S1 — Comparison of parameter estimates of 4 DupliPHY-ML models run on the Génolevures data. (PDF) [file pone.0099480.s007.pdf]

Table 1: Comparison of 4 DupliPHY-ML models run on the Génolevures data.

Model	Birth (b)	Death (d)	Innovation (i)	Extinction (e)	Gamma (g)
BDIE+G	1.00	4.05	0.05	0.59	0.28
BDIE	1.00	4.15	0.48	0.82	-
BDI	1.00	2.80	0.70	-	-
BDI+G	1.00	3.00	0.08	-	0.27
