# Supplementary material for: Inferring Gene Family Histories in Yeast Identifies Lineage Specific Expansions
Source: PLoS One. 2014 Jun 12;9(6):e99480. doi: 10.1371/journal.pone.0099480 (PMC4055711; doi:10.1371/journal.pone.0099480)
Supplement: Table S2 — The effects of gene family data on the estimation of rates under the BDIE+G model. (PDF) [file pone.0099480.s008.pdf]

Table 1: The effects of gene family data on the estimation of rates under the BDIE+G model.

Dataset	Num. families	Ave. size	Birth (b)	Death (d)	Innovation (i)	Extinction (e)	Gamma (g)
Génolevures	4578	9.72	1.00	4.05	0.05	0.59	0.28
Tribe-MCL (I = 1.4)	4888	10.04	1.00	3.23	0.08	0.21	0.36
Tribe-MCL (I = 2)	5352	9.17	1.00	3.81	0.06	0.23	0.37
Tribe-MCL (I = 4)	5763	8.49	1.00	4.53	0.04	0.19	0.34
Tribe-MCL (I = 6)	5870	8.33	1.00	4.69	0.04	0.19	0.34
